# Gamma-aminobutyric acid as a potential postbiotic mediator in the gut–brain axis

**DOI:** 10.1038/s41538-024-00253-2

**Published:** 2024-04-02

**Authors:** Jason D. Braga, Masubon Thongngam, Thanutchaporn Kumrungsee

**Affiliations:** 1 Laboratory of Molecular Nutrition, Graduate School of Integrated Sciences for Life, Hiroshima University, Hiroshima, 739-8527 Japan; 2 Institute of Food Science and Technology, College of Agriculture, Food, Environment and Natural Resources, Cavite State University, Indang, Cavite, 4122 Philippines; 3 Department of Food Science and Technology, Faculty of Agro-Industry, Kasetsart University, Bangkok, 10900 Thailand; 4 Smart Agriculture, Graduate School of Innovation and Practice for Smart Society, Hiroshima University, Hiroshima, 739-8527 Japan

**Keywords:** Applied microbiology, Neuroscience

## Abstract

Gamma-aminobutyric acid (GABA) plays a crucial role in the central nervous system as an inhibitory neurotransmitter. Imbalances of this neurotransmitter are associated with neurological diseases, such as Alzheimer’s and Parkinson’s disease, and psychological disorders, including anxiety, depression, and stress. Since GABA has long been believed to not cross the blood–brain barrier, the effects of circulating GABA on the brain are neglected. However, emerging evidence has demonstrated that changes in both circulating and brain levels of GABA are associated with changes in gut microbiota composition and that changes in GABA levels and microbiota composition play a role in modulating mental health. This recent research has raised the possibility that GABA may be a potent mediator of the gut–brain axis. This review article will cover up-to-date information about GABA-producing microorganisms isolated from human gut and food sources, explanation why those microorganisms produce GABA, food factors inducing gut–GABA production, evidence suggesting GABA as a mediator linking between gut microbiota and mental health, including anxiety, depression, stress, epilepsy, autism spectrum disorder, and attention deficit hyperactivity disorder, and novel information regarding homocarnosine-a predominant brain peptide that is a putative downstream mediator of GABA in regulating brain functions. This review will help us to understand how the gut microbiota and GABA-homocarnosine metabolism play a significant role in brain functions. Nonetheless, it could support further research on the use of GABA production-inducing microorganisms and food factors as agents to treat neurological and psychological disorders.

In the gut, trillions of microbes form a complex community, commonly known as the gut microbiota^1^. Gut microbiota produces thousands of unique small molecules or metabolites that can potentially affect host health^2^. Commonly identified metabolites include short-chain fatty acids (SCFAs), bile acids, choline metabolites^3,4^, vitamins^5^, amino acids^6^, and neurotransmitters^7^. The bidirectional communication pathway between the gut microbiota and the gut and their interaction with the central nervous system is termed the brain–gut–microbiome axis^8^. The gut microbiota and its metabolites affect host health through the brain^8^ and peripheral systems^9^. Metabolites travel by sending signals to the brain via the vagus nerve^10^ or blood–brain barrier (BBB) after crossing the intestinal barrier^11^. These metabolites are considered postbiotics because they can improve disease phenotypes and regulate the gut microbiota and metabolic pathways^12^ (Fig. 1). In contrast, dyshomeostasis of the gut microbiota and postbiotics leads to a variety of diseases in the host, such as metabolic, cardiovascular, and neurological diseases^13,14^.

**Fig. 1:**
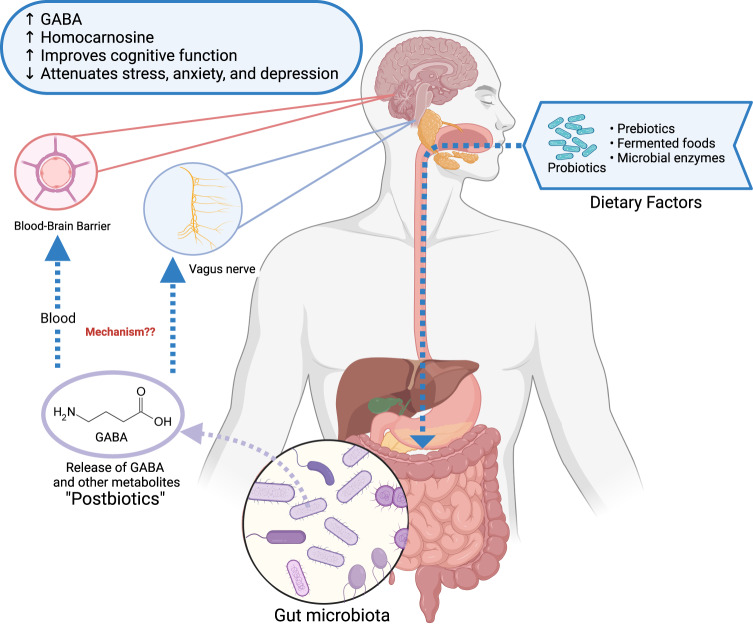
Overview of the interplay of dietary factors, gut microbiota, microbial metabolites, and brain health. These dietary factors, including probiotics, prebiotics, fermented foods, and microbial enzymes, positively affect gut microbiota composition that stimulates the release of GABA and other microbial metabolites. As microbial GABA passes through the intestinal barrier, it influences brain compound levels via blood-brain barrier or vagus nerve and improves brain function. This figure was created using Biorender.com.

Among recently developed postbiotics, GABA has gained much attention. Liu et al. (2017) showed changes in bacteria with the glutamic acid decarboxylase (GAD, K01580) enzyme gene, which is responsible for converting glutamate to GABA between control and obese individuals^15^. Furthermore, Kootte et al. (2017) demonstrated that GABA and GABA-producing bacteria were the most altered plasma metabolites and bacteria in fecal microbiota transplantation from lean individuals to people with metabolic syndrome^16^. Moreover, the intake of probiotics, such as *Lactobacillus* and *Bifidobacterium*, promotes an increase in GABA in both the gut and the brain^11,17,18^. These findings indicate that GABA is a possible postbiotic mediator of the gut–brain axis, which in turn regulates host health.

This manuscript reviews the development of GABA-producing microorganisms isolated from the human gut and fermented food products, as well as their potential to mediate the gut–brain axis based on available scientific evidence.

## GABA metabolism

GABA was first discovered in the brain in 1950^19^; years later, it was recognized as a key inhibitory neurotransmitter^20^. The functional importance of GABA is not limited to the brain; evidence suggests that it also has significance in peripheral tissues such as the gut, urinary bladder, heart, lung, ovary, and pancreas^21^. In terms of GABA concentration, the brain contains a high concentration with an average value of 2–3 mmol/g wet weight (20–30 mmol/g protein) and a regional distribution of 10–30 mmol/g protein, whereas most peripheral tissues have low GABA content, which is approximately 1% of that in the brain^21^. Among peripheral organs, GABA is abundant in the pancreas, and recent research suggests that the pancreatic GABA system plays an important role in protecting the pancreas and regulating insulin metabolism^22^.

### GABA synthesis

GABA is synthesized by various organisms, including humans, plants, and bacteria^23^. In the synthesizing process, GABA is produced from glutamate by the glutamic acid decarboxylase (GAD) enzyme that requires pyridoxal-5′-phosphate (PLP) as a cofactor^24^. GAD enzymes exist in two forms, GAD65 and GAD67, which are regulated by GAD1 and GAD2, respectively^25^. In humans, GAD genes play a crucial role in the brain, where they are involved in the release of the inhibitory neurotransmitter GABA. GAD65 and GAD67 are present in the axon terminals and cell bodies, respectively^26^. GAD65 operates at a small fraction of its maximal catalytic capacity because its activity is very sensitive to changes in the energy state (inorganic phosphate, phosphocreatine, pH, magnesium, adenosine diphosphate (ADP), and adenosine triphosphate (ADP)) and the availability of PLP (an active form of vitamin B6)^27^, which is an allosteric cofactor of GAD enzymes. GAD expression is regulated at the transcriptional and post-translational levels, and it plays a key role in maintaining the balance between glutamate and GABA^28^. In peripheral organs, GAD is highly expressed in the pancreas as both the GAD65 and GAD67 isoforms, similar to its expression in the brain^22^. In plants, GAD is activated by abiotic stress (hypoxia, heat, cold, drought, and mechanical wounding) or biotic stress (predation and infection-induced wounding) to accumulate GABA^29^. In bacteria, GAD expression is induced during stationary or log phase growth under osmotic stress^30^.

### GABA degradation

**Fig. 2:**
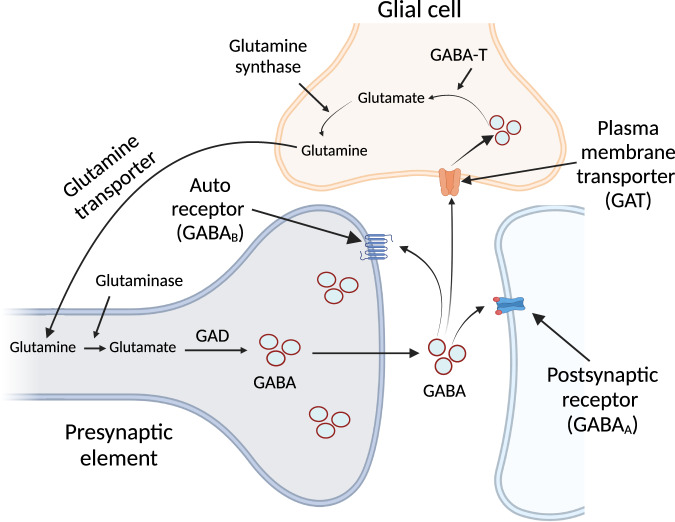
Catabolism and anabolism of GABA. The highly expressed GABA transaminase (GABA-T) from glial cells is responsible for the clearing of released GABA from the synapses to convert GABA into glutamate, which is fed into the glutamine pool. Then, glutamine is transported from the glial cells to the presynaptic element, where it is converted back to glutamate. Then, glutamate is converted to GABA by glutamic acid decarboxylase (GAD). This figure was created using Biorender.com.

In the presence of the GABA-transaminase (GABA-T) enzyme, GABA is catabolized to succinic semialdehyde (SSA) by transamination with the co-substrate of α-ketoglutarate. Subsequently, SSA is oxidized by SSA dehydrogenase (SSADH) to succinate, a constituent of the tricarboxylic acid (TCA) cycle^31^. GABA-T is highly expressed in human glial cells and is responsible for clearing released GABA from the synapses to convert GABA into glutamate, which is then fed into the glutamine pool (Fig. 2)^32^. In addition to its expression in glial cells, GABA-T is also expressed in brain capillary endothelial cells, where it is believed to act as a neurotransmitter-metabolizing enzyme that possibly hydrolyzes circulating GABA and protects it from entering the brain^33^. In peripheral organs, GABA-T is highly expressed in the liver and, to some extent, in the pancreas and kidneys^34^. However, why GABA-T is highly expressed in the liver remains unclear. One hypothesis is that GABA-T hydrolyzes dietary or exogenous GABA and prevents the entry of peripheral GABA into the brain. To support this hypothesis, a previous study demonstrated that 2% GABA mixed in the diet (20 g GABA/kg diet) did not increase blood GABA levels, and even at a high dose (5% GABA), it could increase blood GABA levels to only +2% of the control group^35^. Although it seems likely that peripheral GABA is highly hydrolyzed in the liver, many studies have demonstrated the beneficial effects of low doses of GABA derived from diet or gut bacteria. It would be interesting to investigate this paradox further.

## GABA-producing microorganisms

### GABA-producing microorganisms isolated from the human gut

Several gastrointestinal (GI) bacteria contain the gene encoding GAD^36,37^, which is responsible for GABA production (Table 1). Among the human microbiota, *Bifidobacterium, Lactobacillus, and Bacteroides* are the most well-known GABA producers^36,37^. GABA production by *Bifidobacterium and Lactobacillus* has been extensively studied because of their probiotic functions and the need for probiotic and fermented food development. Emerging evidence has revealed that *Bacteroides* may be the primary genus in gut microbiota influencing mental health through the regulation of GABA production. Compared to *Bifidobacterium and Lactobacillus, Bacteroides* is one of the most abundant and prevalent genera in the human gut microbiota^37,38^. Recent findings from animal and human studies have shown a strong relationship between mental health disorders and dysregulation of the gut microbiota linked to glutamate–GABA metabolism, in which changes in the composition of *Bacteroides* were pronounced in the mental health disorder group^37,39,40^. In this section of the review, we focus only on those that have been reported to produce GABA in humans.

**Table 1:** GABA-producing microorganisms isolated from the human gut

Microorganism	Characteristic	References
*Lactobacillus brevis* DPC6108	Converts 10 and 20 mg/ml of MSG to GABA at 100% conversion rate	^41^
*Lactobacillus brevis* 15 f *Bifidobacterium angulatum* GT102	Efficient GABA producer	^42^
*Bifidobacterium adolescentis* 150	Possess antibiotic-resistant and antioxidant activity	^43^
*Lactobacillus plantarum* 90sk	GABA production is affected PLP addition; possesses antibiotic-resistant and antioxidant activity	^42,43^
*Bacteroides fragilis KLE1758* *Bacteroides caccae KLE1911* *Bacteroides vulgatus KLE1910* *Bacteroides ovatus KLE1170* *Bacteroides dorei KLE1912*	Abundant levels negatively correlated with brain signatures associated with depression	^37^
*Bacteroides* spp.	Regulation of the GABAergic system in the human gut	^36^
*Bifidobacterium adolescentis* PRL2019 *Bifidobacterium adolescentis* HD17T2H	In vivo production of GABA with potential implication in gut–brain axis interactions	^44^

A recent Integrated Microbial Genomes/Human Microbiome Project database showed that *Bacteroides* (31.7%) was the most abundant genus in human gut microbiota processing GAD orthologs (specifically *gadB*), followed by *Escherichia* (22.5%) and *Fusobacterium* (9.9%); and both *Bifidobacterium* and *Lactobacillus* processed only 2.2%^38^. A recent study identifying uncultured bacteria in the human microbiome revealed that GABA is a previously undescribed growth factor in uncultured bacteria, and the main GABA producer is *Bacteroides fragilis*^37^. In addition to *Bacteroides*, *Parabacteroides*, *Eubacterium*, and *Bifidobacterium* have been identified as GABA producers in human stool samples^37^. However, only *Bacteroides* can produce GABA under a physiological pH for the human large intestine (pH 5.7–7.4), in which generally acid-tolerant pathogens such as *E. coli* produce GABA at a lower pH (<5.5)^37,38^. Transcriptome analysis of stool samples from healthy individuals confirmed that *Bacteroides*, *Parabacteroides*, and *E. coli* are GABA producers in the human gut^38^. Subsequently, information on GABA production by *Bacteroides* strains isolated from the human intestine was provided. Eleven species of human-intestinal *Bacteroides*, *B. caccae*, *B. dorei*, *B. faecis*, *B. fragilis*, *B. intestinalis*, *B. ovatus*, *B. plebeius*, *B. thetaiotaomicron*, *B. uniformis*, *B. vulgatus*, and *B. xylanisolvens*i, were found to produce GABA within the range from 0.1 to 61 mM^36^, comparable to GABA levels produced by high GABA-producing *Lactobacillus* and *Bifidobacterium* strains isolated from infant feces (12–300 mM)^41^. Using *B. thetaiotaomicron* as a culture model, it was found that both glutamate and glutamine are substrates for GABA production, and *Bacteroides* can produce GABA at pH values ranging pH from 3.1 to 6.3, with the highest production ability at pH 3.1^36^. In silico analysis using a total of 961 *Bacteroides* genomes revealed that 96% of human–gut isolated *Bacteroides* genomes uniquely harbor four genes, a GAD (gadB ortholog; IPR010107), a glutaminase (glsA ortholog; IPR015868), a glutamate/GABA antiporter (gadC ortholog, IPR022520), and a K+ channel (IPR028325). These genes are involved in the GAD enzyme system^36^ and may exert a protective mechanism against acid stress in *Bacteroides*. *Bacteroides* can adapt to human gut conditions during the host lifespan with the flexibility to use various energy sources from both diet- and host-derived nutrients. This makes them resilient and robust against colonization of the human gastrointestinal tract^36^.

*Lactobacillus* and *Bifidobacterium* are well known for their probiotic effects. Several studies have reported the isolation of *Lactobacillus* and *Bifidobacterium* from the human gut^37,38,41–44^. A previous study demonstrated that GABA can be produced from human fecal fermentation, with GABA concentrations ranging from 5.4 to 56.4 µM^41^. By screening 91 strains from seven species of *Lactobacillus* and 13 species of *Bifidobacterium* isolated from infants and adults, it was found that only one *Lactobacillus* and three *Bifidobacterium* strains could produce GABA; these four GABA-producing strains were isolated from infant feces, dental carriers, and ileocecal junction areas^41^. With 10 mg/mL MSG supplementation, *L. brevis* DPC6108, *B. adolescentis* DPC6044, *B. dentium* DPC6333, *B. dentium* NFBC2243, and *B. infantis* UCC35624, exhibited GABA production at 106.7, 21.3, 50.9, 59.1, and 33.6 mM, respectively^41^. A later study showed that among 135 strains from 13 species of *Lactobacillus* and three species of *Bifidobacterium* isolated from healthy adults, only two species of *Lactobacillus*, *L. plantarum*, and *L. brevis*, exhibited GABA production, while all three species of *Bifidobacterium* (*B. adolescentis*, *B. angulatum*, and *B. dentium*), displayed GABA production^36^. *L. plantarum* (30 strains) and *L. brevis* (three strains) produced GABA at 0.5–2.9 mM and 0.5–6.5 mM, respectively, while *B. adolescentis* (21 strains), *B. angulatum* (three strains), and *B. dentium* (one strain) produced GABA at 4.7–58.2, 25.4–33.6, and 23.9 mM, respectively^42^. The addition of exogenous PLP, a cofactor of GAD, to the culture medium was found to increase GABA production in *L. plantarum* but not in *L. brevis, B. angulatum*, or *B. adolescentis*^42^. Recent research suggests that the *Bifidobacteriaceae* family, together with *Streptococcaceae*, is associated with a higher abundance of fecal GABA in healthy individuals with no systemic or psychiatric illnesses^45^. This high abundance of *Bifidobacterium* is beneficial, especially when alpha diversity in the gut is low (associated with specific diseases) because it restores microbial diversity^46^. The key player responsible for the high abundance of GABA in feces is *Bifidobacterium adolescentis*, which was recently recognized as a gut microorganism involved in GABA production^44,45^. *B. adolescentis* 150, *B. adolescentis* PRL2019, and *B. adolescentis* HD17T2H were bifidobacterial strains that are efficient GABA producers^43,44^. A recent study suggested that some *Bifidobacterium adolescentis* may represent a GABA producer model due to their performance in vitro and in vivo^44^. These *B. adolescentis* strains were identified as *B. adolescentis* PRL2019 and *B. adolescentis* HD17T2H, which can produce 7.1 mM and 9.4 mM of GABA, respectively^44^. By de novo genome assembly, *B. angulatum* GT102 and *B. adolescentis* 150 strains contain *gadB* gene encoding glutamate decarboxylase, *gadС* gene encoding putative glutamate/gamma-aminobutyrate antiporter, and gene encoding monoamine oxidase involved in the catabolism of monoamines^47^. In addition to their GABA-producing ability, both *L. plantarum* 90sk and *B. adolescentis* 150 exhibit antibiotic resistance and antioxidant properties^43^.

In addition to *Bacteroides*, *Lactobacillus*, and *Bifidobacterium*, a recent study demonstrated that *Lactococcus*, a genus of lactic acid bacteria, can produce GABA^48^. *Lactococcus garvieae* MJF010 was found to be the most efficient GABA producer among 23 lactic acid bacteria strains isolated from healthy human feces^48^. The GAD enzyme of *L. garvieae* MJF010 showed the highest GABA-producing activity at 35 °C and pH 5, whereas exogenous PLP addition had no effect^48^.

These studies provide compelling evidence that human gut microbiota is capable of producing GABA and may play a role in mediating gut and host health. Research focusing on other gut microorganisms is of great importance to further understand their critical roles in the gastrointestinal tract. Moreover, commensal probiotic strains in the human gut can be considered delivery vehicles for GABA in specific regions of the gut^49^.

### GABA-producing microorganisms isolated from foods

Extensive studies have been conducted to develop GABA-rich food supplements^50^ and fermented foods^51^ that leverage many health benefits of GABA^52^. Recently, GABA production has been focused on seeking highly productive GABA strains and optimizing the growth conditions of these bacteria^53^. In Japan, the food industry is mainly interested in GABA production because it is considered a bioactive compound that promotes health and can be leveraged in the development of foods for specific health use (FOSHU)^54^.

Fermenting vegetables, meat, and fruits using lactic acid bacteria (LAB) is a standard method for preserving and improving the dietary and sensory characteristics of food commodities^55^. The complex nutritional substances of food commodities are a rich source of vitamins and minerals necessary for the growth of LAB strains, which facilitate the microbial production of enzymes and other metabolites^56^. LAB efficiently and rapidly converts sugars to lactic acid as a primary metabolic product, contributing to the preservation of fermented foods. Many of these raw materials contain significant amounts of glutamate, which can be utilized by LAB to convert glutamate to GABA using the GAD enzyme to increase tolerance to acidic conditions^57^. Several GABA-producing LAB have been isolated from a wide range of fermented foods (Table 2).

**Table 2:** GABA-producing microorganisms isolated from fermented foods and their produced GABA concentration

Microorganism	Source	GABA production	References
*Lactobacillus brevis* HY1	*Kimchi*	18.76 mM	^58^
*Lactobacillus brevis* L-32	*Kimchi*	349.1 mM	^66^
*Lactobacillus brevis* NCL912	*Paocai*	149.05 mM	^69^
*Lactobacillus brevis* BJ-20	*Jeotgal*	0.02 mM	^67^
*Lactobacillus brevis* K203	*Kimchi*	430.57 mM	^135^
*Lactobacillus brevis* 877 G	*Kimchi*	18.94 mM	^136^
*Lactobacillus paracasei* NFRI 7415	Fermented fish	302 mM	^59^
*Lactobacillus plantarum* DM5	*Marcha Sikkim*	NR*	^137^
*Lactobacillus plantarum* DSM19463	Cheese	4.83 mM	^138^
*Lactobacillus plantarum* C48	Cheese	16 mg/kg	^139^
*Lactobacillus paracasei* PF6	Cheese	99.9 mg/kg	
*Lactobacillus brevis* PM17	Cheese	15 mg/kg	
*Lactobacillus lactis* PU1	Cheese	36 mg/kg	
*L. delbrueckii* subsp. *bulgaricus* PR1	Cheese	63 mg/kg	
*Lactobacillus brevis* CECT 8183	Goat cheese	0.96 mM	^53^
*Lactobacillus brevis* CECT 8182	Sheep cheese	0.94 mM	
*Lactobacillus brevis* CECT 8182	Goat cheese	0.99 mM	
*Lactobacillus brevis* CECT 8184	Goat cheese	0.93 mM	
*Lactobacillus helveticus* NDO1	*Koumiss*	1.55 mM	^61^
*Lactobacillus paracasei* 15 C	Raw milk cheese	14.8 mg/kg	^140^
*Lactobacillus rhamnosus* 21D-B	Raw milk cheese	11.3 mg/kg	
*Streptococcus thermophilus* 84 C	Raw milk cheese	11.3 mg/kg	
*Lactobacillus buchneri* WPZ001	Chinese fermented sausage	1250.97 mM	^60^
*Lactococcus lactis* subsp. *lactis* B	*Kimchi* and yoghurt	62.16 mM	^62^
*Streptomyces bacillaris* strain R9	Tea	2.9 mg/kg	^63^
*Streptomyces bacillaris* strain Y11	Tea	2.4 mg/kg	
*Lactobacillus buchneri* MS	*Kimchi*	251 mM	^141^
*Lactobacillus namurensis* NH2	*Nham*	87.86 mM	^68^
*Pediococcus pentosaceus* HN8	*Nham*	71.18 mM	
*Lactobacillus futsaii* CS3	*Kung-som*	242.44 mM	^70^
*Enterococcus faecium* JK29	*Kimchi*	14.86 mM	^142^
*Lactobacillus plantarum* IFK 10	Fermented soybean	25.99 mM	^143^
*Weissella hellenica* SB101	*ika-koujizuke*	69.63 mM	^71^
*Weissella hellenica* SB105	*ika-kurozukuri*	74.57 mM	
*Levilactobacillus brevis* F064A	*Nham*	27.64 mM	^144^
*Lactobacillus brevis* DSM 32386	Traditional Alpine cheese	2.54 mM	^145^
*Pediococcus pentosaceus* ENM104	Fermented ground pork	0.04 mM	^64^
*Lactobacillus plantarum* SPS109	Thai fermented fish	0.04 mM	
*Saccharomyces cerevisiae* SC125	Chinese *Paocai*	9.99 mM	^74^
*Lactobacillus plantarum* BC114	Chinese *Paocai*	14.06 mM	
*Kluyveromyces lactis* BIOTEC009	Mexican milk kefir grain	1.66 mM	^72^
*Lactococcus lactis* BIOTEC008	Mexican milk kefir grain	0.29 mM	
*Weissella paramesenteroides* N-7	Sourdough	18.43 mM	^65^
*Weissella* cibaria N-9	Sourdough	12.32 mM	
*Leuconostoc pseudomesenteroides* N-13	Sourdough	10.20 mM	
*Lactobacillus paraplantarum* N-15	Sourdough	6.49 mM	
*Lactobacillus curvatus* N-19	Sourdough	14.17 mM	
*Lactobacillus rossiae* ED-1	Sourdough	11.04 mM	
*Lactobacillus plantarum* ED-10	Sourdough	15.47 mM	
*Lactobacillus brevis* E-25	Sourdough	11.92 mM	
*Lactobacillus paralimentarius* E-106	Sourdough	3.39 mM	
*Weissella cibaria* SC-20	Sourdough	3.80 mM	
*Leuconostoc citreum* SC-7	Sourdough	4.57 mM	
*Leuconostoc citreum* SC-10	Sourdough	4.92 mM	
*Lactobacillus graminis* SC-12	Sourdough	3.90 mM	
Lactobacillus plantarum SC-9	Sourdough	4.92 mM	
*Leuconostoc mesenteroides* N-6	Sourdough	15.19 mM	

^*^NR – Not Reported.

The predominant species of GABA-producing microorganisms described in Table 2 are *Lactobacillus* spp., including *L. brevis*^58^, *L. plantarum*^50^, *L. paracasei*^59^, *L. buchneri*^60^, and *L. helveticus*^61^. Among these, *Lactobacillus paracasei* NFRI 7415, isolated from fermented fish, produces high levels of GABA (302 mM) under appropriate conditions^59^. GABA-producing microorganisms were isolated from a wide range of fermented foods including cheese^55^, yogurt^62^, tea^63^, ground pork^64^, and sourdough^65^ as well as various Asian fermented products such as kimchi^66^, *jeotgal* (Korean fermented fish)^67^, *nham* (fermented Thai sausage)^68^, *paocai* (Chinese fermented vegetables)^69^, *kung-som* (Thai fermented shrimp)^70^, and *ika-koujizuke* (Japanese squid fermented with malted rice) and *ika-kurozukuri* (Japanese squid fermented with squid ink)^71^.

Recently, microorganisms belonging to the genera *Lactococcus*, *Lactobacillus*, *Leuconostoc*, and *Kluyveromyces* were identified and isolated from Mexican milk kefir grains and showed good probiotic properties through aggregation abilities, antimicrobial activity, antibiotic susceptibility, and resistance to in vitro gastrointestinal digestion, comparable to commercial probiotics^72^. Specific isolates of *Kluyveromyces* (BIOTEC009 and BIOTEC010), *Leuconostoc* (BIOTEC011 and BIOTEC012), and *Lactobacillus* (BIOTEC014 and BIOTEC15) exhibited high fermentability in media supplemented with commercial prebiotics^72^. The capacity to produce GABA was classified as a medium-level GABA producer for *L. lactis* BIOTEC006, BIOTEC007, BIOTEC008, *K. lactis* BIOTEC009, *L. pseudomesenteroides* BIOTEC012, and *L. kefiri* BIOTEC014 and was comparable to that obtained for commercial probiotics^72^. The classification system for GABA production by microorganisms was adapted from Tsukatani et al. (2005): less than 0.5 mM was considered a low-level GABA-producer, 0.5–2.1 mM was a medium-level GABA-producer, and more than 2.1 mM was a high-level GABA-producer^73^. Moreover, *Saccharomyces cerevisiae* SC125 and *Lactobacillus plantarum* BC114, both isolated and identified from traditional Chinese *paocai*, yielded 23.5 mM GABA as a co-culture that promotes the production of flavor compounds and GABA in mulberry beverage brewing^74^.

Despite the great diversity of fermented food products available worldwide as products of various cultures and traditions, little is known about the microorganisms involved in the fermentation process. There may be undiscovered microorganisms in traditional fermented products that are more efficient producers of GABA and other compounds than those previously identified and documented. Moreover, commercially available fermented products, such as *kimchi*, provide more data and information on the microorganisms involved in the fermentation process. Hence, a focus on other traditional fermented products is necessary to help diversify the information on fermentation and the potential of fermenting microorganisms to produce GABA and other beneficial metabolites.

## Why do microorganisms produce GABA?

**Fig. 3:**
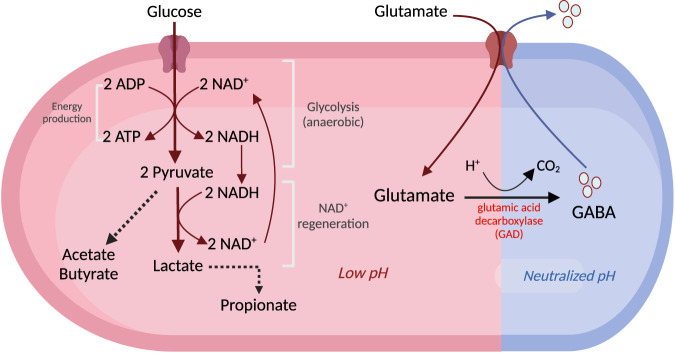
Mechanism of GABA production in microorganisms. Under anaerobic and acidic conditions in the human gut and fermentation, it appears that bacteria produce GABA for their own survival purposes under these extreme environments. Under anaerobic conditions, glycolysis takes place in the cytosol, where NAD^+^ and ADP are required to convert glucose into pyruvate, in which NADH and ATP are produced from the process^75^. Pyruvate is then converted into lactate or other organic compounds, such as acetate, butyrate, and propionate, where NADH is utilized, and NAD^+^ is generated in the process. Then, NAD^+^ is fed back and reutilized in the glycolysis process^75^. The acidic fermentation by-products, lactate, and other organic compounds lower the pH, which leads bacteria to utilize the GAD gene system and triggers the production of GABA^76,77^. To produce GABA, exogenous glutamate is transported into the cell by a glutamate/GABA antiporter, then glutamate is converted into GABA by glutamic acid decarboxylase (GAD)^36,76,78^. Then, GABA is exported from the cell via the antiporter, resulting in an increase in the pH of the cytoplasm due to the removal of H^+^ ions and a slight increase in the extracellular pH due to the exchange of extracellular glutamate for more alkaline GABA^76,78^. This figure was created using Biorender.com.

It is common knowledge in this research field that bacteria, especially those with probiotic properties, can produce GABA because of their ability to express GAD genes. However, the reason by which these bacteria produce GABA remains unclear. It has been hypothesized that GABA is produced under anaerobic and acidic conditions, allowing bacteria to survive in extreme environments. As shown in Fig. 3, glycolysis takes place in the cytosol under anaerobic conditions, where two molecules each of nicotinamide adenine dinucleotide (NAD^+^) and ADP are required to convert a glucose molecule into two molecules each of pyruvate nicotinamide adenine dinucleotide + hydrogen (NADH), and ATP^75^. Next, pyruvate is converted into fermentation products such as lactate, ethanol, and organic compounds, including acetate, butyrate, and propionate, in which two NAD^+^ molecules are generated, fed back, and reutilized in the glycolysis process^75^. The production of lactate and other acids by bacteria during fermentation lowers the pH, which leads to bacteria utilizing the GAD gene system^76^. The GAD gene system, consisting of *gadB*, glutaminase, *gadC* (glutamate/GABA antiporter), and K^+^ channels, helps bacteria cope with changes in intracellular pH^36^. Activation of the GAD gene system owing to a decline in pH then triggers the production of GABA^76,77^. As shown in Fig. 3, free glutamate in the environment is transported into the cell by a specific transporter (glutamate/GABA antiporter), which leads to the decarboxylation of glutamate to GABA by GAD, in which intracellular H^+^ ions are consumed^36,76,78^. GABA is then exported from the cell via the antiporter, which results in an increase in the pH of the cytoplasm due to the removal of H^+^ ions and a slight increase in the extracellular pH due to the exchange of extracellular glutamate for more alkaline GABA^76,78^. Hence, the release of GABA helps bacteria cope with acid stress, which is crucial for the colonization of the GI tract and survival in acidic fermentation environments.

## Gut–GABA-production inducing food factors

In addition to fermented food products that promote GABA production owing to the presence of GABA-producing bacteria, several researchers have explored the potential of other food factors that can induce GABA production in the gut. As mentioned above, probiotics, *Bifidobacterium* and *Lactobacillus*, and the predominant gut bacteria *Bacteroides* are the main GABA producers in the human gut, and food factors that can enhance the abundance of these gut bacteria are candidates for GABA production-inducing food factors. In addition to typical well-known prebiotics, such as fructooligosaccharides (FOS), emerging research suggests that microorganism-derived enzymes, such as proteases, lipases, amylases, and cellulases, have the potential to act as prebiotics to increase probiotics in the gut^79–82^. Recent studies have shown that dietary *Aspergillus oryzae*-derived protease markedly increases the abundance of both *Bifidobacterium* and *Lactobacillus* in the rat cecum and induces the production of various postbiotics, including GABA, which was not detected in the cecum of rats receiving no dietary protease^80,81^. Taken together with the fact that GABA is a non-proteinogenic amino acid, these findings suggest that GABA was possibly produced from elevated levels of the probiotics *Bifidobacterium* and *Lactobacillus*. More recent studies have revealed that other dietary factors, such as lipase from *Penicillium camemberti*, which is generally used in cheese production, also induce an increased abundance of *Bifidobacterium* and *Lactobacillus* in the rat cecum^83^. These studies imply that the digestive enzymes produced by *Aspergillus* and *Penicillium* exert prebiotic-like effects by increasing the abundance of the GABA-producing probiotics *Bifidobacterium* and *Lactobacillus*, in the gut, possibly making them efficient in GABA production. The same is true for inulin, which stimulates GABA production in the gut^84^. More work is needed to identify and investigate other food factors that have the potential to induce GABA production in the gut or brain, regardless of whether they are microorganism-derived or naturally derived.

## Gaba as a mediator of the gut–brain axis

### Association of gut microbiota and GABA in mental health and brain function

It has been well-accepted that dysbiosis of the gut microbiota is strongly linked to human health, including mental health. Gut microbiota and probiotics impact host health through various mechanisms, including the production of metabolites, recently defined as postbiotics, such as short-chain fatty acids, peptides, and amino acids. Among these postbiotics, GABA has received much attention from researchers owing to its essential role in the nervous system and its strong correlation with the gut microbiota. Studies have suggested that peripheral or circulating GABA is mainly attributed to the gut microbiota^40,85,86^. In germ-free mice, blood (3.7 times) and fecal (1.3 times) GABA levels were lower than those in fecal-oral-inoculated germ-free mice^85^. Another study showed that cecal GABA levels were markedly decreased in mice treated with vancomycin^86^. Oral administration of GABA-producing *B. dentium ATCC 27678*, but not non-GABA-producing *B. breve* NCIMB8807, increased cecal GABA levels and reduced colon-specific sensory neuron excitability, which are the general causes of abdominal pain^38^. Taken together, these studies indicate the potential role of GABA as a moderator in the gut–brain axis. The following section presents recent information regarding the involvement of the gut microbiota and GABA in mental health and brain diseases. A summary of this interaction is shown in Table 3.

**Table 3:** Summary of the association between gut microbiota, GABA, and brain diseases

Diseases	Gut microorganism	Influence on GABA	References
Schizophrenia	↑*Bacteroidaceae* ↑*Coriobacteriaceae* ↑*Prevotellaceae* ↑*Veillonellaceae* ↓*Lachnospiraceae* ↓*Norank* ↓*Ruminococceae*	↑GABA levels	^88^
	↑Eggerthella* ↑Escherichia/Shigella* ↑*Lactobacillus* ↑*Megasphaera* ↑*Prevotella* ↑*Veillonella* ↓Bacteroides* ↓*Coprococcus* ↓*Haemophilus* ↓*Roseburia* ↓Streptococcus*		^89^
Alzheimer’s disease	↑Atopobiaceae* ↑*Clostridiales* ↑*Erysipelotrichaceae* ↑Lachnospiraceae* ↑*Prevotellaceae* ↑*Pseudomonadaceae* ↑Ruminococceae* ↑*Synergistaceae* ↓*Erysipelotrichaceae* (*Erysipelatoclostridium*) ↓Lachnospiraceae* (*Tyzzerella*)	↓N-docosahexaenoyl GABA	^90^
	↑*Porphyromonadaceae* ↓*Blautia* ↓*Erysipelotrichaceae*	↓GABA levels	^92^
Parkinson’s disease	↑*Akkermansia* ↓*Lactobacillus* ↑*Erysipelotrichaceae* ↑*Enterococcaceae* ↓*Muribaculaceae* ↓*Lachnospiraceae* ↓*Defluviitaleaceae*	↓GABA levels	^94,96^
Anxiety and stress	↑*Faecalibacterium prausnitzii* ↑*Collinsella aerofaciens* ↑*Flavonifractor sp*. An100 ↑*Victivallis vadensis* ↑*Ruminococcaceae bacterium* ↓ *Eubacterium rectale* ↓*Megamonas funiformis* ↓*Lactobacillus rogosae* ↓Bacteroides eggerthii* ↓*Acidaminococcus intestine* ↓*Paraprevotella clara* CAG:116 ↓*Lachnospiraceae bacterium* AM48-27BH	↓GABA synthesis	^99^
Depression	↑*Flavonifactor plautii* ↑*Pseudomonas* spp. ↑*Acinetobacter* spp.	↑GABA degradation	^98^
Epilepsy	↓*Anaerotruncus* ↓*Peptococcaceae* ↓*Prevotellaceae Ga6A1* group ↓*Ruminococcus torques* group ↓*Peptococcus y Ruminococcus gauvreauii* group ↓Pseudomonas graminis* ↓*Ruminococcaceae bacterium* AM2	↓GABA levels	^102^
	↓*Akkermansia muciniphila* ↓*Parabacteroides*	↓GABA/glutamate levels	^105^
Autism spectrum disorder	↑Clostridium* ↑*Klebsiella* ↓Bifidobacterium* ↓*Prevotella copri* ↓*Feacalibacterium prausnitzii* ↓*Haemophilus parainfluenzae*	↓GABA levels	^108,109^
	↑Escherichia/Shigella* ↑*Lachnoclostridium* ↑*Megamonas* ↑Megasphaera* ↑Veillonella* ↓*Bacteroides* ↓*Akkermansia* ↓*Parabacteroide* ↓*Rothia*	↑GABA/glutamate levels	^110^
	↑*Dialister* ↑*Escherichia/Shigella* ↑Bifidobacterium* ↓*Prevotella 9*	↑GABA precursor levels	^111^
Attention deficit hyperactivity disorder	↑*Bacteroides* ↑*Dorea* ↑*Erysipelotrichaceae* ↑*Ruminococcaceae* ↑*Dialister* ↓*Lachnospiraceae* ↓*Ruminococcus* ↓*Bacteroides* ↓*Lachnospiraceae* ↓*Enterococcus*	↓GABA levels (putative)	^114^
	↑*Bifidobacterium adolescentis* ↑*Bifidobacterium animalis* ↑*Bifidobacterium breve* ↑*Bifidobacterium longum* ↑*Bacteroides ovatus* ↑*Bacteroides uniformis* ↑*Fusobacterium ulcerans* ↑*Enterocococcus avium* ↑*Enterococcus gallinarumi* ↓*Faecalibacterium prausnitzii*	↑GABA levels (putative)	^115^
	↓*Lactobacillus rogosae* ↓*Lactobacillus ruminis*		

↑ indicates increasing; ↓ indicates decreasing; * indicates bacteria that were reported to have direct impact on GABA metabolism.

### Gut microbiota and GABA in neurological disorders

Neurological disorders such as schizophrenia (SCZ), Alzheimer’s disease (AD), and Parkinson’s disease (PD) have been linked to dysbiosis because of the strong connection between the gut and brain^87^. A recent study revealed that treated and non-treated SCZ patients had a decreased microbiome diversity index compared to healthy controls, where an increased abundance of *Veillonellaceae*, *Prevotellaceae*, *Bacteroidaceae*, *Coriobacteriaceae* and a decreased abundance of *Lachnospiraceae*, *Ruminococceae*, and *Norank* were found in SCZ patients^88^. Additionally, a lower abundance of *Bacteroides* and *Streptococcus* in the gut microbiota is a feature of SCZ, and these bacteria are associated with glutamate and GABA metabolism^89^. Furthermore, germ-free mice receiving the SCZ microbiome showed decreased glutamate but increased GABA levels in the hippocampus, displaying SCZ-relevant behaviors similar to other mouse models of SCZ involving glutamatergic hypofunction^88^. In AD, the fecal microbial composition and metabolic output were evident. Patients with AD had an increased abundance of *Lachnospiraceae*, *Ruminococcaceae*, *Prevotellaceae*, *Atopobiaceae*, *Clostridiales*, *Synergistaceae*, *Erysipelotrichaceae*, and *Pseudomonadaceae* and a decreased abundance of *Lachnospiraceae* (genus *Tyzzerella*) and *Erysipelotrichaceae* (genus *Erysipelatoclostridium*) compared to normal controls, and these microorganisms were significantly associated with a decreased abundance of N-docosahexaenoyl GABA, 19-oxoandrost-4-ene-3,17-dione, trigofoenoside F, and 22-angeloylbarringtogenol C metabolites^90^. Bidirectional Mendelian randomization analysis has revealed a causal relationship between the relative abundance of *Blautia*, a new functional genus with potential probiotic properties^91^, and AD^92^. Elevated levels of GABA, a downstream product of *Blautia*-dependent arginine metabolism, in the cerebrospinal fluid (CSF) are related to a reduced risk of AD^92,93^. Patients with PD had a significant increase in *Akkermansia* and a decrease in *Lactobacillus* compared to healthy controls^94^. The differences in postural instability gait difficulty (PIGD) and tremor-dominant (TD) PD motor subtypes in basal ganglia GABA levels could be lower in TD than in PIGD, which may indicate a difference in the pathophysiological mechanisms of TD and PIGD^95^. In addition, treatment with *Pediococcus pentosaceus* improved the gut microbial dysbiosis and increased GABA levels in methyl-4-phenyl-1,2,3,6-tetrahydropyridine (MPTP)-induced PD^96^.

### Gut microbiota and GABA in anxiety, depression, and stress

Recently, altered gut microbiota and reduced function of the GABA system in the prefrontal cortex following chronic ethanol exposure led to anxiety-like behaviors^97^. Administration of *Lactobacillus rhamnosus* JB-1 improved stress-induced anxiety- and depression-like behaviors in mice by increasing GABA mRNA expression in the hippocampus^10^. Increased small intestine GABA level (0.03–0.04 mM) of metabolic syndrome mice model fed with diet incorporated with *Lactobacillus brevis* DPC6108 and DSM32386 strains improved depression-like behavior in the forced swim test and resting stress hormone corticosterone level compared to high-fat control diet^18^. Metagenomics-based analyses involving datasets collected from children with subclinical symptoms of depression and anxiety revealed high metagenomic reads of *gad* in groups with a high abundance of *Bifidobacterium adolescentis*^44^. Furthermore, the depressed phenotype had a greater prevalence of GABA-consuming microorganisms in the selected strains of *Flavonifractor plautii*, *Pseudomonas* spp., and *Acinetobacter* spp. then the healthy phenotype, thereby favoring GABA degradation^98^. Moreover, a decreased abundance of *Bacteroides eggerthii* was found to be associated with a decrease in GABA synthesis in subjects with stress and anxiety, and gut microbiota modulation through probiotic supplementation enriched GABA-synthesizing *Bifidobacterium adolescentis* and *Bifidobacterium longum* that alleviated stress- and anxiety-related symptoms^99^.

### Gut microbiota and GABA in epilepsy

An imbalance in neuroactive compounds, including GABA, and intestinal dysbiosis are two important considerations in epilepsy^100,101^ and are commonly observed in humans and dogs^102^. In humans, it was found that patients with four or fewer seizures per year had higher fecal *Bifidobacteria* and *Lactobacilli* than those who had more than four seizures^103^. These flora promote GABA synthesis^36,37^. In dogs, the epileptic group had a significantly reduced abundance of fecal GABA-producing (*Pseudomonadales*, *Pseudomonadaceae*, *Pseudomonas*, and *Pseudomona_graminis*) and SCFA-producing bacteria (*Peptococcaceae*, *Ruminococcaceae* and *Anaerotruncus*), as well as bacteria associated with a reduced risk of brain disease (*Prevotellaceae*) compared to the control group^102^. Despite difficulties with implementation, dietary compliance, and adverse side effects, a ketogenic diet (or low-carbohydrate, high-fat diet; KD)^104^ is an effective dietary intervention to treat epilepsy. KD positively altered the gut microbiota by increasing the abundance of *Akkermansia muciniphila* and *Parabacteriodes* from 4 to 14 days of treatment, demonstrating an anti-seizure effect in a wide-range anti-epileptic drug-resistant seizure model^105^. Administration of the KD paired with *Akkermansia muciniphila* and *Parabacteriodes* significantly increased hippocampal GABA/glutamate ratios^105^. Probiotic administration (several *Lactobacillus*, *Bifidobacterium*, and *Streptococcus* strains) to drug-resistant epileptic (DRE) patients decreased the number of seizure occurrences and increased the serum GABA concentration after a 12-week treatment^106^.

### Gut microbiota and GABA in autism spectrum disorder (ASD) and attention deficit hyperactivity disorder (ADHD)

Occurrence of high gut *Clostridium* spp. is associated with ASD in patients with gastrointestinal disease^107^. Specifically, 76–87% of beta2-toxin-producing *Clostridium perfringens* were significantly higher in children with ASD compared to control children, indicating that these opportunistic pathogens thrive in immature or compromised immune systems^107^. A recent study has shown that infants with increased-likelihood of ASD have a decreased abundance of *Bifidobacterium* but an increased abundance of *Clostridium* and *Klebsiella* compared to those with lower likelihood of ASD^108^. Moreover, fecal GABA levels of infants with increased likelihood of ASD were lower than those with lower likelihood of ASD, in which fecal GABA levels are positively correlated with *Bifidobacterium*, but negatively correlated with *Clostridium*^108^. A lower abundance of *Prevotella copri*, *Feacalibacterium prausnitzii*, and *Haemophilus parainfluenzae* and decreased concentrations of fecal GABA were found in children with ADS when compared to healthy children^109^. In contrast, an increased ratio of fecal GABA/glutamate with a higher abundance in *Escherichia/Shigella* and a lower abundance of *Bacteroides* was found in mild ADS children than in healthy children^110^. *Dialister*, *Escherichia/Shigella*, and *Bifidobacterium* were more abundant in ASD children, while *Prevotella 9*, *Megamonas*, and *Ruminococcus 2* were more abundant in healthy children, in which GABA precursors, such as N-carboxyethyl-g-aminobutyric acid, glutamylproline, pyroglutamic acid, and gamma-glutamylglycine, were higher in ASD children^111^.

In ADHD, magnetic resonance spectroscopy revealed a significant reduction in brain GABA concentration in children with ADHD^112^. In contrast, increased cortical GABA concentration was observed in adults with ADHD, which suggests that GABA levels may be correlated with the age of patients with ADHD^113^. A recent study has shown that the top five depleted bacteria families in infants (6 months of age) with ADHD are *Lachnospiraceae*, *Ruminococcus*, *Bacteroides*, *Lachnospiraceae*, and *Enterococcus*, while the top five enriched bacteria families are *Bacteroides*, *Dorea*, *Erysipelotrichaceae*, *Ruminococcaceae*, and *Dialister*^114^. Interestingly, 50% of the depleted families belong to the *Lactobacillales* order, or lactic acid bacteria^114^. Due to the fact that lactic acid bacteria are strong GABA producers, this can suggest that the depletion of lactic acid bacteria in the gut of infants with ADHD might be related to a decrease in GABA. On the other hand, in a case study, an adult with ADHD was found to have a high abundance of *Bifidobacterium adolescentis*, *Bifidobacterium animalis*, *Bifidobacterium breve*, *Bifidobacterium longum*, *Bacteroides ovatus*, *Bacteroides uniformis*, *Fusobacterium ulcerans*, *Enterocococcus avium*, and *Enterococcus gallinarumi*, but fecal microbiota transplant significantly reduced the abundance of those bacteria with the relief of ADHD symptoms^115^. Since most of those bacteria (*Bifidobacterium*, *Bacteroides*, and *Enterocococcus*) are well-known GABA producers^37^, this may support a positive correlation of GABA with ADHD in adults as previously reported^113^. It seems likely that GABA may play a role in the pathogenesis of ADHD in children and adults, but possibly in different ways.

## Brain-specific GABA-containing peptide

### Homocarnosine

Homocarnosine (GABA-l-histidine) is a GABA-containing dipeptide that is predominantly found in the brain^116^. It is an analog of the predominant muscle dipeptide carnosine (β-alanine-l-histidine). Homocarnosine is synthesized from GABA and histidine by carnosine synthase in neurons and is degraded by carnosinase^117,118^. The occipital cortex, basal ganglia, and cervical cord have the highest human homocarnosine synthetase, currently known as carnosine synthase, activity while the cerebellar cortex has the lowest^117^. It is present in greater amounts in the human brain than in the brains of other mammals^119^. Homocarnosine concentration of the autopsied brain ranges from 0.4 mmol/kg in the corpus callosum and temporal cortex to 1.0 mmol/kg in the thalamus and basal ganglia and varies independently of GABA concentrations^120^. The homocarnosine concentration is threefold to sixfold higher in adults than in infants^121^. Recently, areas of the human central nervous system, particularly the olfactory bulb, spinal cord, medulla oblongata, thalamus, cerebellum, white matter, and frontal cortex, had a considerable amount of homocarnosine while the human CSF abundantly contains homocarnosine^122^. Although its concentration in the brain is high, the function of homocarnosine in the brain remains underexplored, which has led to a limited understanding of its high maintained concentrations in the brain. However, several biochemical properties of homocarnosine have been reported.

For instance, homocarnosine acts as a protective agent against a wide range of disease conditions, including the protection of brain endothelial cells from amyloid peptide-induced toxicity^123^ and anti-inflammatory action in brain ischemic injuries^124^. Homocarnosine demonstrates similar properties to carnosine in protecting Cu and Zn superoxide dismutase from oxidative damage through a combination of copper chelation and peroxyl radical scavenging^125^. Moreover, homocarnosine, in combination with carnosine and anserine, reduces oxidative damage by decreasing lipid peroxidation and increasing antioxidant levels in the brain^126^. Many studies have explored the biological role of homocarnosine in the brain and other neurological diseases. Hence, a thorough investigation is required to better understand the role of homocarnosine.

Because homocarnosine is a GABA-containing peptide, changes in GABA levels may contribute to changes in homocarnosine homeostasis. This hypothesis is supported by the notion that homocarnosine is a possible GABA reservoir, as approximately 40% of GABA measured in human CSF is homocarnosine^127^. In addition, it has been hypothesized that the release of homocarnosine contributes to glutamate–GABA cycling and reflects an adaptive response to excess extracellular glutamate^128^, wherein a strong linear correlation between GABA and homocarnosine concentrations has been observed in healthy CSF (GABA concentration is higher than homocarnosine concentration)^129^.

### Possible link of gut–brain axis and GABA-homocarnosine in brain-related diseases

Homocarnosine homeostasis in the brain plays a critical role in clinical studies of neurological diseases, such as Alzheimer’s disease and epilepsy^130,131^. Low homocarnosine levels may reflect decreased fractional volumes of homocarnosine-containing neurons, and homocarnosine deficits may indicate either the loss or dysfunction of GABAergic neurons^128,130^. Drugs may be administered to improve homocarnosine levels in the brain. Vigabatrin and gabapentin, known antiepileptic drugs, improve seizures by increasing levels of brain GABA and homocarnosine^131,132^. Topiramate, another anti-seizure drug, improves brain homocarnosine and GABA levels, contributing to its potent anti-epileptic action in patients with complex partial seizures^127^. Moreover, isoniazid supplementation in healthy patients elevates homocarnosine and GABA concentrations^133^. As mentioned above, homocarnosine could possibly be a good reservoir of GABA in the brain, and other neurological disorders, such as AD, ASD, and SCZ, can be associated with homocarnosine as they are characterized by low GABA levels, and GABA can induce homocarnosine production^35,88,92,134^.

It is worth mentioning that the above-mentioned neurological disorders alter gut GABA-producing microorganisms that affect GABA homeostasis in the gut and brain. An increase in the abundance of the well-known probiotics *Lactobacillus* and *Bifidobacterium* induces gut GABA production^79,80^. However, to date, the direct interaction and correlation between homocarnosine and the gut microbiota as affected by diet remain unknown. Recently (unpublished data), our group discovered the ability of *Aspergillu*s-derived enzymes together with FOS to exhibit a tendency to increase cecal and brain GABA levels. Moreover, the dietary intake of these prebiotic-linked enzymes and FOS increases homocarnosine levels in the brain. These findings indicate that dietary factors may act as one of the modulators of GABA and homocarnosine levels in the gut and brain.

To summarize, GABA has long been the subject of rigorous research, and its health benefits have been proven through in vitro and in vivo experiments. Although circulating GABA has long been believed to not cross the BBB, GABA’s permeability through the BBB remains contested due to conflicting evidence. Recent research has demonstrated that GABA can be a potent mediator of the gut–brain axis, as it is circulating and brain levels are regulated by the microbiota, and that changes in GABA levels and microbiota composition play a role in modulating mental health. Generally, GABA is present at trace concentrations in the bloodstream. Recent studies have suggested that circulating GABA is mainly attributed to gut microbiota. Several studies have isolated GABA-producing bacteria from the human gut, such as *Lactobacillus*, *Bifidobacterium*, and *Bacteroides*, and from fermented foods, such as *Lactobacillus, Streptococcus*, *Leuconostoc*, and *Weisella*. In addition to probiotics, non-typical prebiotic food factors, such as *Aspergillus*- and *Penicillium*-derived enzymes, have been demonstrated to stimulate an increased abundance of probiotics and gut GABA production. Supplementation with probiotics and probiotic-rich products improves the cognitive function of patients with neurological disorders; eases anxiety, depression, and stress; and increases circulating and brain GABA availability. In addition to GABA, a predominant GABA-containing brain peptide, homocarnosine, has recently been demonstrated to be a possible downstream mediator of GABA in the gut–brain axis. Currently, there is limited information regarding the connections between homocarnosine, gut microbiota, and brain function. Thus, it is of great importance to further investigate this issue because this information may help clarify how the gut microbiota and GABA-homocarnosine metabolism play a role in brain function. This information will contribute to the development of functional foods and mental health interventions.

### Reporting summary

Further information on research design is available in the Nature Research Reporting Summary linked to this article.

### Supplementary information

Reporting summary
